# Endogenous phenotype of diagnostic transition from major depressive disorder to bipolar disorder: a prospective cohort study

**DOI:** 10.1192/j.eurpsy.2024.675

**Published:** 2024-08-27

**Authors:** S. Shim

**Affiliations:** psychiatry, soonchunhyang university cheonan hospital, cheonan, Korea, Republic Of

## Abstract

**Introduction:**

This study investigated sensor-level electroencephalography (EEG) power and related source-level cortical activity using resting-state EEG in patients with MDD and BD.

**Objectives:**

This study aims to comparing bipolar disorder (BD) and major depressive disorder (MDD) to understand neuropathology of these disorders.

**Methods:**

A total of 68 patients with MDD were enrolled and recorded EEG. Among patients with MDD, 17 patients with MDD converted to BD during the study periods. Clinical symptoms and EEG measures were compared between two groups. This study applied machine learning to differentiate the two groups using sensor and source-level features

**Results:**

At the sensor level, patients with BD showed higher power of AF3 channel in the theta beta band(p=0.011) and FC5 channel in the low alpha band(p=0.014), compared to MDD. At the source-level, compared to MDD, patients with BD showed higher activity in the right anterior cingulate(p=0.011) and left parahippocampal gyrus(p=0.035). The best classification performance for MDD and BD showed an accuracy of 80.88%, a sensitivity of 76.47%, and a specificity of 82.35% based on theta and low alpha band power and activity features.

**Conclusions:**

Our findings might suggest different theta and low alpha band activity between patients with BD and MDD might serve clinically as a candidate neuromarker for differentiating two distinct mood disorders.

**Disclosure of Interest:**

None Declared

